# MicroRNA-184 negatively regulates corneal epithelial wound healing via targeting *CDC25A*, *CARM1*, and *LASP1*

**DOI:** 10.1186/s40662-020-00202-6

**Published:** 2020-08-01

**Authors:** Qiongjie Cao, Weiwei Xu, Weiwei Chen, Dewei Peng, Qi Liu, Jing Dong, Peter S. Reinach, Dongsheng Yan

**Affiliations:** 1 School of Ophthalmology and Optometry, Eye Hospital, Wenzhou Medical University, 270 Xueyuan Road, Wenzhou, 325027 Zhejiang China; 2 State Key Laboratory of Ophthalmology, Optometry and Visual Science, Wenzhou, Zhejiang China

**Keywords:** miR-184, Corneal epithelial wound healing, Proliferation, Migration, CDC25A, CARM1, LASP1

## Abstract

**Background:**

MicroRNAs (miRNAs) play critical roles in corneal development and functional homeostasis. Our previous study identified miR-184 as one of the most highly expressed miRNAs in the corneal epithelium. Even though its expression level plummeted dramatically during corneal epithelial wound healing (CEWH), its precise role in mediating corneal epithelial renewal was unresolved. The present study aimed to reveal the function and mechanism of miR-184 in regulating CEWH.

**Methods:**

Quantitative RT-PCR analysis characterized the miR-184 expression pattern during CEWH in mice. Ectopic miR-184 injection determined its effect on this process in vivo. We evaluated the effects of miR-184 and its target genes on the proliferation, cell cycle, and migration of human corneal epithelial cells (HCECs) using MTS, flow cytometry, and wound-healing assay, respectively. Bioinformatic analysis, in conjunction with gene microarray analysis and cell-based luciferase assays, pinpointed gene targets of miR-184 contributing to CEWH.

**Results:**

MiR-184 underwent marked downregulation during mouse CEWH. Ectopic miR-184 overexpression delayed this process in mice. Furthermore, miR-184 transfection into HCECs significantly inhibited cell proliferation, cell cycle progression, and cell migration. MiR-184 directly targeted *CDC25A*, *CARM1*, and *LASP1*, and downregulated their expression in HCECs. CARM1 downregulation inhibited both HCEC proliferation and migration, whereas a decrease in LASP1 gene expression only inhibited migration.

**Conclusions:**

Our results demonstrate that miR-184 inhibits corneal epithelial cell proliferation and migration via targeting *CDC25A*, *CARM1*, and *LASP1*, suggesting it acts as a negative modulator during CEWH. Therefore, identifying strategies to suppress miR-184 expression levels has the potential to promote CEWH.

## Background

Continuous epithelial renewal of the cornea is essential for maintaining tissue transparency and vision. This replacement process preserves cell to cell tight junctional integrity, which provides a protective barrier function against pathogenic infiltration into the inner tissue layers [1, 2]. In addition, the renewal process sustains a smooth optical surface needed for supporting the refractive power of the eye. Epithelial renewal is under the control of a host of cytokines regulating cell proliferation, migration, and differentiation [3–6]. Control of each of these function stems in part from their modulation of gene expression levels. If severe injury or infection disrupts this renewal process, pathogenic infiltration into the stroma can occur and disrupt its organization and makeup. Such changes can lead to tissue swelling and opacification, followed by losses in visual acuity [7]. In order to improve the treatment of sight compromised by epithelial wounds in a clinical setting, numerous studies are underway to clarify how these mediators modulate gene expression during wound healing [8, 9]. Through these endeavors, it may be possible to identify novel drug targets for reducing the risk of corneal infection and complications, possibly requiring transplantation surgery.

MicroRNAs (miRNAs) are small non-coding, single-stranded RNAs that are about 22 nucleotides in length, and they act as repressors of gene expression. They regulate in a sequence-specific manner gene expression via base pair binding to the 3′-untranslated region (3′-UTR) or 5′-UTR of target mRNAs. The extent of nucleotide base complementarity between a miRNA and an mRNA target determines whether such interaction leads to mRNA degradation or translation termination [10]. In the corneal epithelium, miRNAs control the expression of gene products that in turn affect lineage specification, cell migration, neovascularization, and proliferation along with cell survival and differentiation as well as glycogen storage [11]. One approach to delineating how miRNAs elicit control of the responses underlying epithelial wound healing is to associate differences in their expression patterns with functional activity.

Despite the involvement of an abundant number of different types of miRNAs in corneal epithelial renewal, miR-184 attracted our attention because it is the most abundant miRNA in this tissue [12]. Furthermore, it is selectively enriched in the mouse lens epithelium, human retinal pigment epithelial cells, and zebrafish lens epithelium [13–16]. It was first shown to act as an antagomir of miR-205 expression in corneal epithelial cells [17]. More recently, other studies demonstrated an involvement for miR-184 in controlling lineage specification, severe keratoconus and cataract formation, as well as corneal angiostasis and glycogen storage. Its suppression of angiogenesis stems from its inhibition of the AKT and VEGF signaling pathway axes [11].

We previously identified 600 candidate miRNAs whose expression levels are affected by injury. It is interesting that only miR-204 and miR-184 were dramatically downregulated more than 200-fold in the proliferating corneal epithelial cells that were positioned behind the leading edge of a wound [12]. However, the association between declines in miR-184 expression and increases in corneal epithelial cell proliferation is somewhat controversial because it was reported that changes in miR-184 levels were unrelated to S-phase progression in the cell cycle [18]. Nevertheless, there are other reports suggesting an involvement for changes in miR-184 in mediating control of responses underlying corneal epithelial cell renewal [19]. Interestingly, miR-184 is uniformly distributed across the basal epithelial cells and immediate suprabasal cells in the unwounded murine cornea, but it is completely absent from the peripheral proliferating limbal cells and adjoining conjunctival epithelium as well as all other ocular tissues. As with miR-184, miR-204 expression levels are downregulated in the proliferating cells located behind the quiescent migrating cells at the leading edge of a wound [18]. The higher expression level of miR-204 in the less proliferative epithelial cells agrees with the fact that miR-204 can lead to cell cycle arrest and inhibition of corneal epithelial cell proliferation [12]. Despite the aforementioned results suggesting that miR-184 is an essential regulator of corneal epithelial functions, neither the precise function nor its gene targets mediating control of corneal epithelial wound healing (CEWH) are known.

We describe here the effects of miR-184 on cell proliferation and migration in mouse CEWH and human corneal epithelial cell (HCEC) models. Microarray and bioinformatic analysis identified a cohort of potential gene targets whose modulation affects responses underlying corneal epithelial renewal. Three of the miR-184 targets that could provide a means for improved therapeutic control of epithelial wound healing are *CDC25A*, *CARM1*, also known as *PRMT4* (PRMT, Protein Arginine Methyl Transferase), and *LASP1*. This study also shows that miR-184 can serve as a biomarker during CEWH and a potential drug target in promoting this process.

## Methods

### In vivo corneal epithelial wound healing model

Eight-week-old C57BL/6 mice were purchased from Vital River Laboratory Animal Technology Co., Ltd. (Beijing, China) and maintained in a climate-controlled environment with a 12-h day/night cycle. Mice had ad libitum access to food and water. All animal experiments were carried out in strict accordance with the ARVO Statement for the Use of Animals in Ophthalmic and Vision Research. The study was approved by the Laboratory Animal Ethics Committee of Wenzhou Medical University (ID Number: wydw2019–0385). Intraperitoneal injections of ketamine 87 mg/kg and xylazine 13 mg/kg body weight were used to anesthetize the mice prior to the procedure. An equal number of male and female mice were included. Next, thirty mice eyes were locally anesthetized with a drop of proparacaine hydrochloride eye drops (Alcon, Inc., Puurs, Belgium). The entire thickness of the corneal epithelium was removed up to the corneal/limbal border using a 0.5-mm corneal rust ring remover (AlgerBrush II, The Alger Company, Inc., Lago Vista, TX, USA). Erythromycin eye ointment was applied to the wounded eyes immediately after scraping to prevent bacterial infection. Healing continued until approximately 10% of the wounded area remained, which took about 48 h. At various time points, the whole corneal epithelia from both the contralateral uninjured and injured corneas were collected for RNA extraction.

### Intrastromal MiR-184 ectopic transfection in wound healing assay

Prior to performing the aforementioned wound healing procedure, sixteen C57BL/6 mice were anesthetized, and the eyes were made accessible for intra-stromal injection. A 33-gauge needle that was attached to a 5 μL syringe (Hamilton, Bonaduz, Switzerland) was inserted into the corneal stroma underlying the limbal area. Next, 1 μL (100 μM) miR-184 mimic (Cat#: 4464066; Thermo Fisher Scientific, Waltham, MA, USA), 0.1 μL 100 g/ml polyethyleneimine (PEI, The Polyplus transfection company, NY, USA) and 2 μL 10% glucose solution were injected. The fellow eye provided a negative control (NC) because it was injected instead with an irrelevant oligonucleotide (Cat#: 4464058; Thermo Fisher Scientific). Six hours post-injection, epithelial scraping was performed as previously described. At 0 and 24 h after the scraping, sodium fluorescein was used to stain the wound area. The area of epithelial defect was imaged with a Leica MZ7.5 microscope and measured by ImageJ software (1.46r; Wayne Rasband, National Institutes of Health, Bethesda, MD, USA). The measurements were conducted by two independent observers and averaged within each group. The percentage of wound closure (%) was evaluated by normalizing the healing area to the initial wound area at 0 h.

### Cell culture and transfection

The SV40-immortalized HCECs (gift from Araki Sasaki Kagoshima, Miyata Eye Clinic, Kagoshima, Japan) were kept in DMEM/F12 medium (Thermo Fisher Scientific) that was supplemented with 10% fetal bovine serum (Thermo Fisher Scientific), 10 ng/mL insulin (Sigma-Aldrich, St. Louis, MO, USA), and 10 ng/mL epidermal growth factor (EGF, Sigma) in a humidified incubator at 37 °C with 5% CO_2_. HEK-293 cells were grown in DMEM supplemented with 10% fetal bovine serum under the same conditions. HCECs were seeded onto 6-, 24-, or 96-well plates (Corning, Inc., Corning, NY, USA) 1 day before the transfections were performed. Cells were transfected with either 50 nM miR-184 mimic for miR-184 overexpression, or a NC, using a transfection reagent (Lipofectamine RNAiMAX; Thermo Fisher Scientific). For CARM1 or LASP1 knockdown, cells were transfected with either 50 nM siRNA against CARM1 or LASP1 or a NC. Synthetic siRNAs were purchased from Sigma-Aldrich (Supplementary Table S1).

### Quantitative RT-PCR (RT-qPCR)

Total RNA was extracted by the Trizol Reagent (Thermo Fisher Scientific) and its integrity was measured by NanoDrop ND-1000 spectrophotometer (Thermo Fisher Scientific). Quantitative RT-PCR was used to evaluate the miR-184 expression levels with a TaqMan MicroRNA Assay (Applied Biosystems, Foster City, CA, USA) on a real-time PCR system (7500 Fast Real-Time PCR System; Applied Biosystems). Small nuclear U6 snRNA expression was used to normalize the relative expression of miR-184. For mRNAs, 1 μg of total RNA was used for cDNA synthesis with a Reverse Transcription System (Promega, Madison, WI, USA). Diluted cDNA was then employed as a standard for quantitative RT-PCR, which was performed with SYBR Green PCR Master Mix (Thermo Fisher Scientific). mRNA expression was normalized to the level of glyceraldehyde-3-phosphate dehydrogenase (GAPDH). All the experiments were performed in triplicates.

### Cell proliferation and flow Cytometric assay

Forty-eight hours after transfection, cell proliferation was assessed with an assay kit (CellTiter 96 Aqueous; Promega) according to the manufacturer’s instructions. HCECs cultured in 6-well plates were stained with propidium iodide (PI) using the Cycle Test Plus DNA Reagent Kit (Becton Dickinson, San Jose, CA, USA) for flow cytometric analysis. The cells were then analyzed for DNA content with flow cytometry (FACS Caliber; Becton Dickinson). All the experiments were performed in triplicates.

### In vitro scratch wound assay

HCECs at approximately 60% confluence were transfected with miR-184 or NC. The cell layers were scratched using a pipette tip after 24 h and were then cultured in fresh serum-free medium. After culture, the cell-free area was measured and normalized based on photographs taken immediately after scraping, at 0 h, and again at 24 h (Imager Z1; Zeiss, Jena, Germany) using Image J software. All the experiments were performed in triplicates.

### Luciferase reporter assays

Luciferase gene reporter assay was used to validate potential miR-184 target identity. *CDC25A*, *CARM1*, and *LASP1* 3′-UTRs and their mutant 3′-UTRs were amplified from human cDNA with PCR using specific primer pairs. Seed regions were mutated from UCCGUCC to AGGCAGG, removing all complementarity to 7 nucleotides of miR-184 by using the QuickchangeXL Mutagenesis Kit (Stratagene, La Jolla, CA, USA). Mutant and WT inserts were confirmed with sequencing. They were inserted into the pMIR-REPORT vector (Thermo Fisher Scientific), downstream from the stop codon of the luciferase gene. The constructs were co-transfected into HEK-293 cells with 50 nM miR-184 mimic or NC using Lipofectamine 2000 reagent (Thermo Fisher Scientific). The relative luciferase activities were detected 24 h after transfection with the Dual Luciferase Reporter System (Promega). All assays were performed in triplicates.

### Western blot analysis

Total protein extracts were collected at 48 h after transfection and processed using standard procedures for Western Blot analysis. The expression levels of CDC25A, CARM1, and LASP1 protein in the cell lysates were quantified using primary antibodies (anti-CDC25A, anti-CARM1; Cell Signaling Technology, Beverly, MA, USA; anti-LASP1; Abcam, Cambridge, UK) with 1:1000 dilution. The endogenous control for normalization was done with an anti-GAPDH antibody (1:1000 dilution; Cell Signaling Technology). The protein bands were quantified with the Image J software. All the experiments were performed in triplicates.

### Microarray and data analyses

We used a Human Transcriptome Array 2.0 (Affymetrix, California, USA) for profiling differential gene expression in 500 ng total RNA extracted from HCECs 48 h after transfection with miR-184 mimic or NC, according to the user manuals. We used the Affymetrix® Command Console Software (Affymetrix) to analyze the microarray data with default settings. Raw microarray data were normalized with Expression Console (Affymetrix) following the quality assessment procedure. The microarray data met the MIAME criteria and have been stored in the National Center for Biotechnology Information (NCBI) Gene Expression Omnibus (GEO). Data can be accessed through http://www.ncbi.nlm.nih.gov/geo (accession number: GSE148289). To analyze the functions of the predicted target genes of miR-184, we exported these predicted genes from TargetScanHuman 7.1 (Whitehead Institute for Biomedical Research, USA). Functional enrichment analysis of the predicted target genes of miR-184 was conducted using the Gene Ontology (GO) Database (http://www.geneontology.org/).

### Statistical analysis

The means and SEM were processed using SPSS 17.0 statistical software (IBM, NY, USA). Data analysis was performed with a two-tailed Student’s *t*-test. *P*-values < 0.05 were considered as statistically significant. Asterisks (*), (**) and (***) indicate *P* < 0.05, *P* < 0.01 and *P* < 0.001.

## Results

### MiR-184 Downregulation during murine corneal epithelial wound healing

**Fig. 1:**
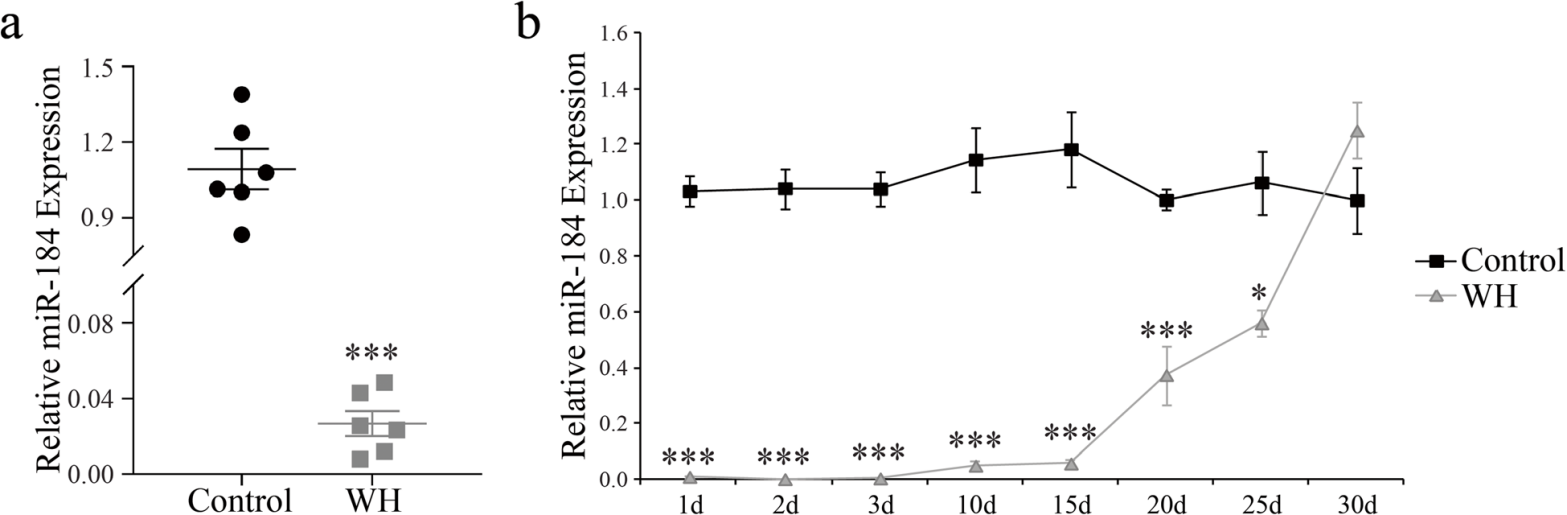
Dramatic miR-184 decline during mouse corneal epithelial wound healing process. **a** Murine corneal epithelia were scratch wounded and collected at 48 h after scraping for analysis. This group was designated as the “wound healing” (WH) group, and the contralateral eye, which was uninjured, was designated as the “Control” group. MiR-184 expression levels were examined with RT-qPCR, and U6 snRNA was used as an internal control (*n* = 6/group). **b** Quantification of miR-184 expression levels in murine corneal epithelia collected at various time points of the wound healing process (*n* = 3/group)

We previously found that only miR-204 and miR-184 were dramatically downregulated over 200-fold during the mouse CEWH process using NanoString nCounter technology [12]. To confirm the changes of miR-184 expression during CEWH, we first determined the miR-184 levels at 48 h after corneal epithelial wounding. RT-qPCR analysis confirmed that the miR-184 expression levels underwent such a substantial decline in murine corneal epithelia relative to that in control uninjured corneas (Fig. 1a). As shown in Fig. 1b, the miR-184 expression levels were remarkably decreased at 24 h after injury and reached a minimum at 48 h. Subsequently, miR-184 expression gradually increased at 96 h to reach the baseline level at 30 days.

### Restoration of miR-184 delays corneal epithelial wound healing in vivo

**Fig. 2:**
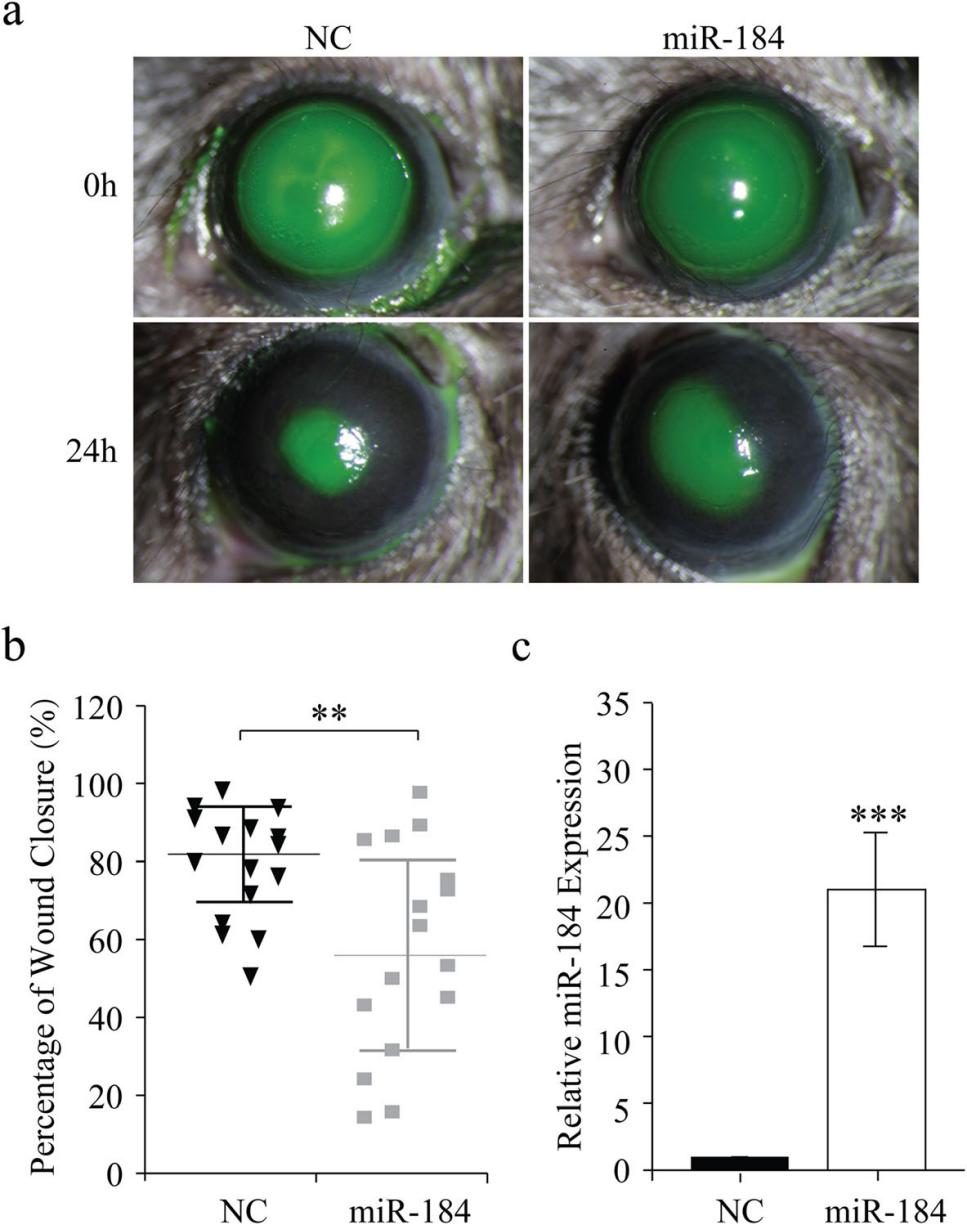
Ectopic miR-184 transfection delays corneal epithelial wound healing. **a** C57BL/6 J mice were injected in the stromal periphery beneath the limbus with miR-184, PEI, and 10% glucose solution. In the fellow eye, the injection contained the negative control (NC). Epithelial scraping was performed 6 h post-injection and epithelial wound areas were observed by staining with sodium fluorescein after 24 h. Wound edges are demarcated in white. **b** The percentage of wound closure (%) was evaluated by normalizing the healing area to the initial wound area at 0 h (*n* = 16/group). **c** RT-qPCR analysis evaluated miR-184 expression level in each murine eye (*n* = 16/group)

To ascertain that such a decline of miR-184 is requisite for CEWH, we determined if miR-184 ectopic expression would delay the wound healing response. MiR-184 mimic was injected into the stroma underlying the limbal area of mice prior to performing the aforementioned wound healing procedure. To visualize the wound healing time dependence, we photographed the sodium fluorescein stained eyes and measured the remaining unhealed areas. MiR-184 gain of function reduced migratory activity relative to the fellow eye treated instead with its irrelevant NC (Fig. 2a). After 24 h, the mean percentage of the healed area was 57.7% in the miR-184 transfected group, which was significantly smaller than the 82.0% in the NC group (*n* = 16, Fig. 2b). This difference suggests that miR-184 upregulation slows the CEWH process. To confirm this notion, we then harvested the scraped corneal epithelium with a corneal rust ring remover and determined the miR-184 expression levels by RT-qPCR analysis. Indeed, this difference in the percentage of wound closure was associated with a much higher miR-184 expression level in the miR-184 transfected tissue than that in the fellow eyes transfected instead with the NC (*n* = 16, Fig. 2c).

### Ectopic miR-184 represses human corneal epithelial cell proliferation and migration

**Fig. 3:**
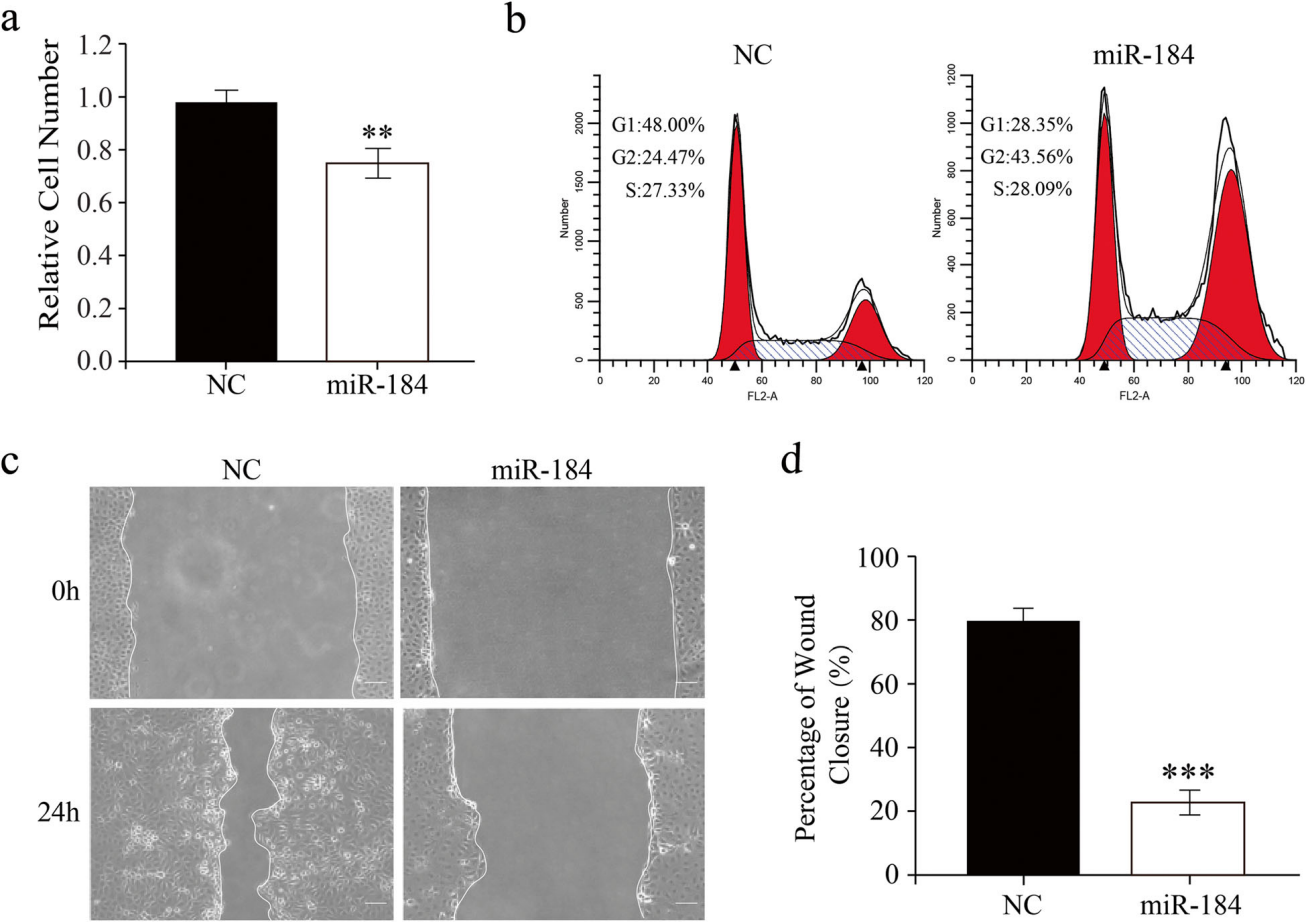
Ectopic miR-184 transfection suppresses human corneal epithelial cell (HCEC) proliferation and migration. **a.** MTS cell proliferation assay was performed at 48 h after transfection with either a miR-184 mimic or negative control (NC). The miR-184 transfected cell number was significantly decreased as compared with the NC (*n* = 3/group). **b.** G2/M phase arrest in the HCEC cell cycle was induced by ectopic miR-184 transfection. HCECs gathered at 48 h after transfection were then stained with PI and analyzed with flow cytometry. **c.** miR-184 transfection reduced HCEC migration. HCECs were transfected with miR-184 or NC for 24 h, and scratches were then made in the HCEC cultures to perform the wound-healing assay. Following culturing for either 0 or 24 h in serum-free DMEM/F12, representative photos of the scratched regions were taken by phase-contrast microscopy. **d.** The percentage of wound closure (%) was quantified by normalizing the healing area to the initial wound area (*n* = 3/group)

Wound healing rates mainly depend on changes in cell proliferation and migration. The effects of ectopic miR-184 expression on these responses in HCECs were determined. The MTS assay was applied after 48 h to determine the effects of HCEC transfection with either a miR-184 mimic or NC on cell proliferation. MiR-184 transfection significantly inhibited proliferation compared with that in control (Fig. 3a). Consistent with this decline, the distribution pattern of HCEC in three cell cycle phases was changed. In the NC transfected cells, their percentage in the G2/M phase was 24.5%, whereas it increased to 43.6% in miR-184 transfected cells (Fig. 3b). The scratch-wound assay was carried out on serum-starved HCECs to assess whether miR-184 upregulation also inhibits HCEC wound closure by suppressing cell migration. MiR-184 transfected cells migrated slower than NC transfected cells within 24 h of wounding since the cultures of miR-184 transfected HCECs had larger wound areas (Fig. 3c-d). This agreement between the effects of miR-184 gain of function in vivo and in vitro on wound healing confirms that its downregulation is requisite for promoting cell proliferation and migration during this process.

### MiR-184 targets *CARM1*, *LASP1*, and *CDC25A*

**Fig. 4:**
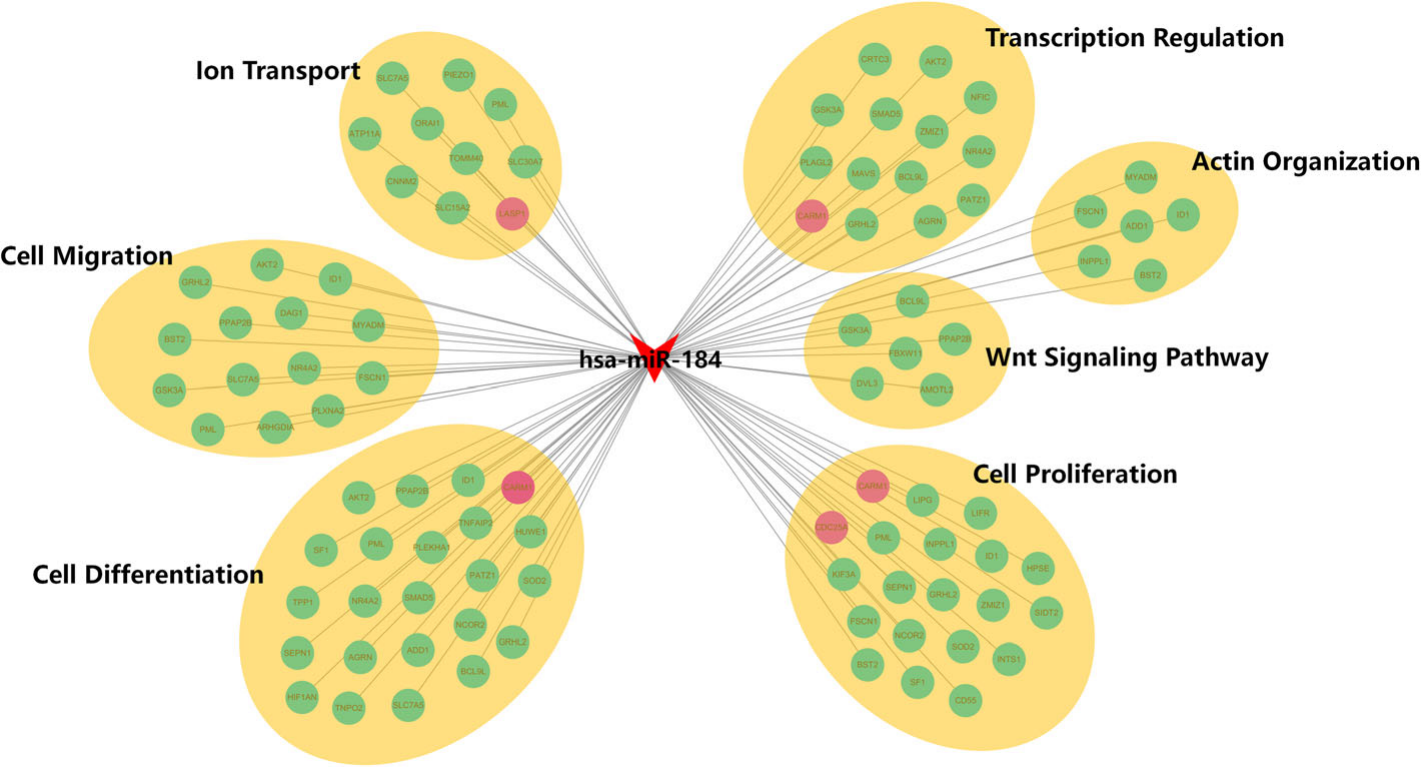
MiR-184 predicted targets and miR-184-target network. Genes that are associated with several GO-terms were chosen to create a network based on a GO enrichment analysis of the predicted target genes for human miR-184. They were also down-regulated in miR-184 transfected human corneal epithelial cells compared with that in the negative control (NC). Red circles represent inserted target genes of miR-184: *CARM1*, *CDC25A*, and *LASP1*

To gain insight into the underlying mechanisms of miR-184 modulation of corneal epithelial cell proliferation and migration, we probed for its gene targets. Microarray analysis and cell-based gene reporter luciferase assays were performed in parallel. HCECs transfected with miR-184 or NC for 48 h were harvested and a gene microarray evaluated transcriptome expression patterns. Ectopic miR-184 expression resulted in numerous genes undergoing either upregulation or downregulation (refer to GSE148289). Since miRNAs mediate biological functions by impeding expression of their target genes in mammals, a total of 901 differentially downregulated genes (Foldchange ≤0.667 and *P* < 0.05) were firstly selected. On the other hand, TargetScan7.1 was conducted for miR-184 target prediction. A total of 104 differentially downregulated genes, which are also putative target genes of miR-184, were then selected for GO enrichment analysis (Supplementary Table S2). Since the wound healing process is under the control of a host of mediators regulating cell proliferation, migration, and differentiation, we focused our attention on those involved in cell proliferation, migration, ionic transport as well as the Wnt signaling pathway (Supplementary Table S3). Figure 4 shows a network of putative miR-184 gene targets based on the GO enrichment analysis. Amongst the functional groupings of these targets, we screened six different representative genes involved with the control of proliferation, migration, ion transport, or transcription groups to validate this analysis. Three genes were identified as direct targets of miR-184: *CDC25A*, required for cell cycle initiation, and *CARM1*, encoded a vital enzyme catalyzing arginine methylation. Another gene, *LASP1*, has essential roles in cell structure, physiological processes, and cell signaling.

**Fig. 5:**
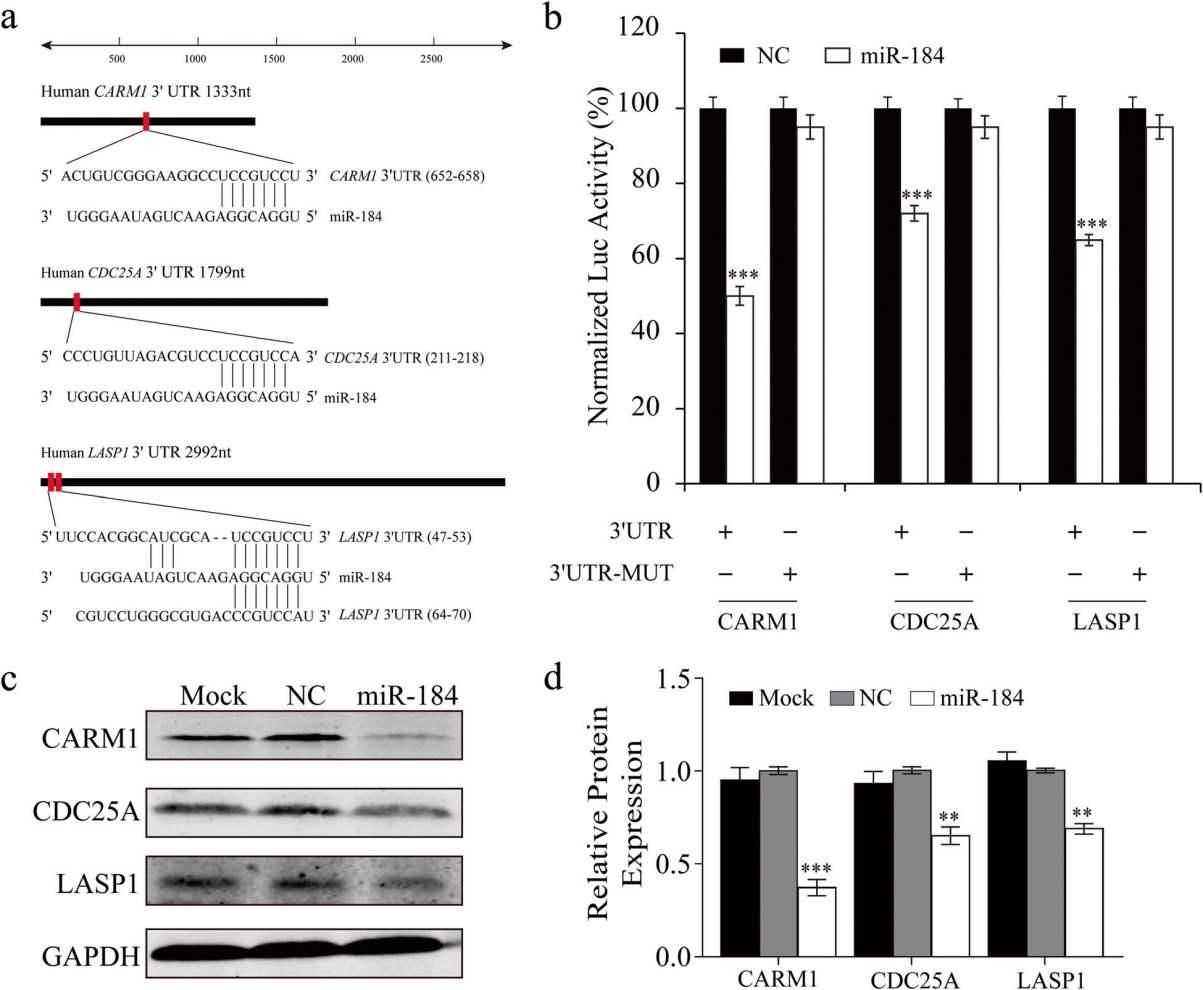
MiR-184 downregulates CARM1, LASP1, and CDC25A expression in HCECs. **a.** Putative miR-184 binding sites in *CARM1*, *LASP1*, and *CDC25A* 3′-UTR. Specific binding sites are shown in bracket pairs next to their gene symbol. Alignment between miR-184 and the predicted miR-184 targets. Their conserved 7-bp seed sequence is shown for miR-184:mRNA pairing. **b.** HEK-293 cells were co-transfected with the expression vectors that were constructed – a pRL-SV40 reporter plasmid and miR-184 mimic or NC. After 24 h, relative luciferase activities were measured. Results are indicated as relative luciferase activity normalized to *Renilla* luciferase activity (*n* = 3/group). **c.** CARM1, LASP1, and CDC25A protein levels determined by Western Blot analysis decreased in HCECs after transfection with miR-184. Ectopic miR-184 expression significantly reduced the levels of CARM1, LASP1, and CDC25A in HCECs compared to the NC. The internal control was GAPDH. **d.** Densitometric Western Blot analysis quantifying the protein expressions of CARM1, LASP1, and CDC25A was performed (*n* = 3/group)

Bioinformatic analysis was carried out on various complementary sequences in the 3′-UTR of these three genes (Fig. 5a). To determine if these candidate genes are targeted by miR-184, the entire wildtype 3′-UTR of each of the target genes was cloned into a luciferase gene reporter vector, respectively. Subsequently, HEK-293 cells were transfected with the same reporter construct along with either miR-184 or NC. At 24 h post-transfection, the luciferase gene reporter assay results showed that its activity was suppressed with each of the three different mRNA constructs (Fig. 5b). However, single nucleotide substitutions within all of the binding sites in the 3′-UTR mitigated the suppression of the activity caused by interaction with miR-184 (Fig. 5b). To verify the specificity of such interaction of either miR-184 or its NC with their predicted targets, Western Blot analysis evaluated corresponding effects on these gene products. Compared to the NC, ectopic miR-184 expression significantly reduced the levels of CARM1, LASP1, and CDC25A in HCECs (Fig. 5c-d). These results confirm that miR-184 can directly target the *CDC25A*, *CARM1*, and *LASP1* genes through binding to their 3′-UTR.

### Downregulation of CARM1 and LASP1 inhibits HCEC proliferation and/or migration

**Fig. 6:**
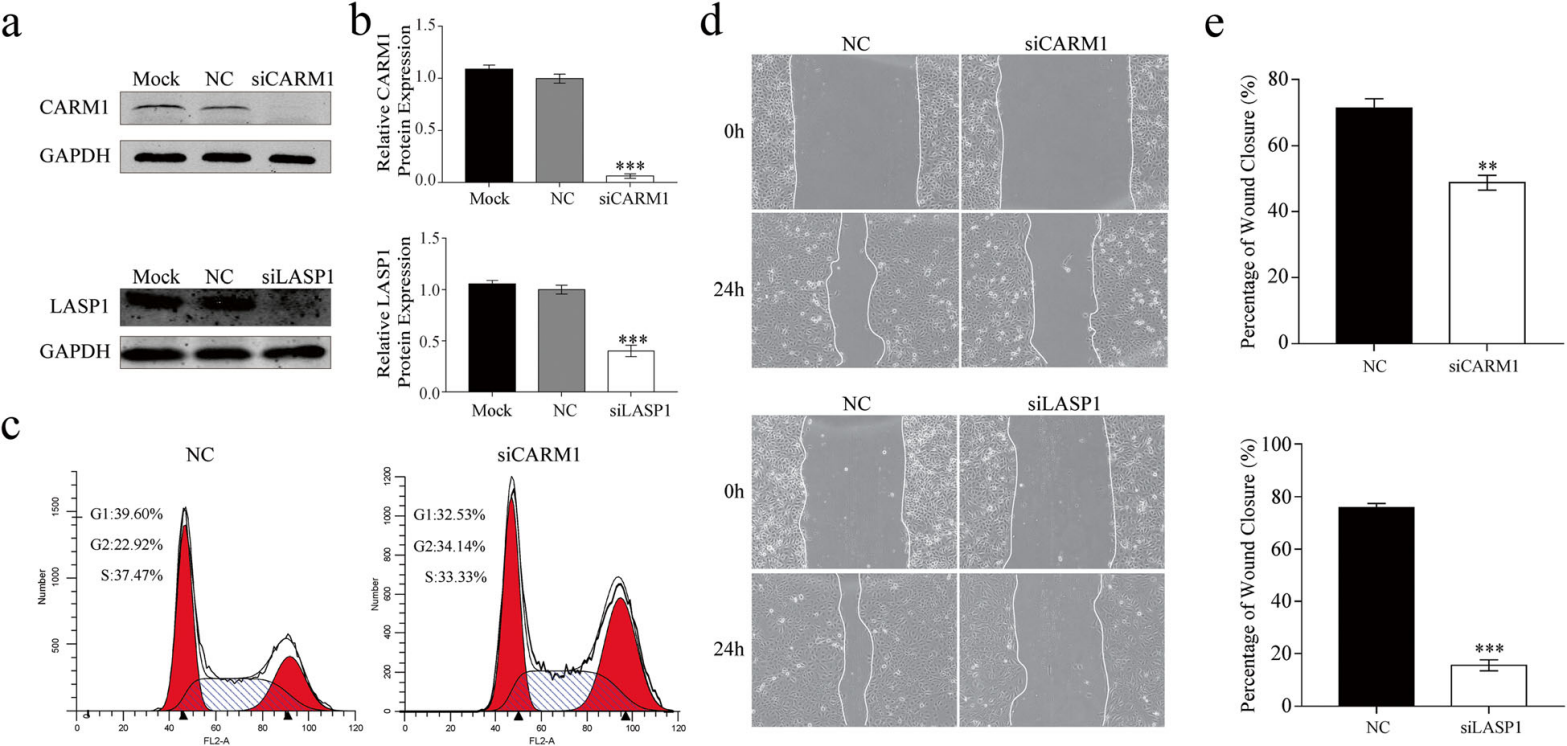
The effects of CARM1 and LASP1 downregulation on human corneal epithelial cell (HCEC) proliferation and migration. **a.** Reductions in CARM1 and LASP1 protein expression levels caused by gene silencing obtained with siRNAs were confirmed by Western Blot analysis. **b.** Densitometric Western Blot analysis showing the difference in protein levels between the negative control (NC)- and the siRNA-transfected groups (*n* = 3/group). **c.** CARM1 knockdown induced G2/M phase arrest in HCECs. HCECs collected at 48 h after transfection with either siCARM1 or NC were stained with PI and analyzed with flow cytometry. **d.** Knockdown of CARM1 or LASP1 inhibited the migration of HCECs. Representative photos of the scratched regions were taken by phase-contrast microscopy. **e.** The percentage of wound closure (%) was quantified by normalizing the healing area to the initial wound area in NC- and the siRNA-transfected groups (*n* = 3/group)

Since *CDC25A*, *LASP1*, and *CARM1* are direct targets of miR-184, we determined whether these targets had similar effects on cell proliferation and migration. As CDC25A is a known regulator of the cell cycle [20], we focused on the cellular function of CARM1 and LASP1 in HCECs. We first used the RNAi technique to knock down the expression of CARM1 and LASP1 in HCECs, and then performed functional analysis. The results shown in Fig. 6a-b indicate that each of these siRNA transfections was effective since their target expression levels decreased significantly. CARM1 downregulation by specific siRNA induced HCEC cell cycle arrest at the G2/M phase (Fig. 6c), consistent with the inhibitory effect of miR-184 transfection on cell cycle progression in HCEC. Furthermore, either LASP1 or CARM1 siRNA transfection both significantly hampered HCEC migration (Fig. 6d-e). Taken together, the underlying mechanism of miR-184 effects on HCEC can be attributed to, at least in part, negative regulation of its targets – *CDC25A*, *CARM1*, and *LASP1*.

## Discussion

Emerging evidence points to important roles of miRNAs in controlling corneal development and diseases [11, 12]. Our previous study on miRNA expression signatures, using NanoString nCounter technology, indicated that miR-204 and miR-184 were the most dramatically downregulated miRNAs during the CEWH process in mice [12]. The dramatic decline in miR-184 expression was noteworthy because it is one of the most abundant corneal epithelial miRNAs; however, neither its precise function nor its mode of action in this process was clear. Besides acting as an antagomir for miR-205, other studies indicated that miR-184 mediates the control of specific gene expression through direct binding to the complementary sequence at 3′-UTRs and affecting translation of genes involved in controlling lineage specification, severe keratoconus and cataract formation as well as angiostasis and glycogen storage [11, 15, 21]. Even though the aforementioned results showed that miR-184 is an essential regulator of corneal epithelial functions, neither the signaling pathways nor its gene targets mediating control of CEWH were known. We validated altered miR-184 expression in the murine CEWH process. Furthermore, we provided convincing evidence to show that a miR-184 expression level decline is requisite for CEWH based on showing that miR-184 ectopic injection significantly delayed this process in vivo. Nevertheless, the underlying mechanism regulating miR-184 expression in wound repair is still unclear. Studies have shown that DNA methylation is involved in regulating miR-184 expression [22–24]. On the other hand, our previous study demonstrated that DNA hypermethylation is responsible for miR-200a downregulation during CEWH [25]. Whether DNA methylation contributes to miR-184 silencing during this process is an interesting topic and warrant further investigation.

The common and high level of miR-184 expression in ocular and cancerous tissues is consistent with its involvement in controlling a host of variable essential cell functions. In squamous carcinoma cells, miR-184 acts as an antagomir of miR-205 and thereby suppresses increases in proliferation induced by miR-205 through suppressing its maintenance of SHIP-2 levels [17]. Similarly, our results indicate that miR-184 negatively regulated the wound healing process through inhibition of corneal epithelial cell proliferation and migration. On the other hand, miR-184 has been found to promote the proliferation of neural stem cells [22] and the migration of epidermal keratinocytes [26]. Therefore, miR-184 acts as an oncogenic modulator in tongue squamous cell carcinoma cell lines [27], which is different from its negative effects on *Drosophila* germline cell proliferation [28] or some other human tumor cells [29]. Even though it was reported that miR-184 was unrelated to S-phase progression in the cell cycle in the murine wound healing process [18], our data showed that miR-184 overexpression reduced HCEC proliferation by cell cycle arrest at the G2/M phase. Such differences indicate a diverse tissue-specific role of miR-184 in controlling cell cycle progression. Besides cell proliferation and migration, differentiation is critical during CEWH [1, 3]. However, it is technically challenging to decipher the role of miR-184 in the differentiation process using primary limbal stem cells. In this study, we focus on miR-184 regulation of cell proliferation and migration as a first step. We intend to explore the involvement of miR-184 in differentiation in future research.

A handful of different genes have been verified as miR-184 targets, including *AKT2*, *BCL2*, *EZR*, *c-MYC*, *FIH1*, *Numbl*, *AGO2*, *PPAP2B*, *PDGF-b*, and *FOG2* [16, 30, 31]. We also identified hundreds of other putative miR-184 target genes based on the results of in silico analysis, which are also down-regulated genes by miR-184 overexpression in HCECs. This constellation includes a total of 104 genes that were used to develop a distinct miR-184-target network, comprised of representative functional GO terms such as Wnt signaling pathway, cell proliferation, differentiation, migration, ion transport as well as transcription regulation. Although analytical methods are insufficient in establishing the reliability and validity of their involvement in controlling cellular homeostasis, in vivo functional analysis can clarify their contribution to this process. Our data further confirmed that *CDC25A*, *CARM1*, and *LASP1* are bona fide targets of miR-184. These findings provide valuable information for further investigation into identifying the molecular mechanism of miR-184 regulation in epithelial wound healing.

MiR-184 can inhibit cell proliferation and invasiveness via targeting *CDC25A* in small lung cell carcinoma [20]. *CARM1*, encoded an essential protein arginine methyltransferase mediating arginine methylation, was experimentally validated as one direct target of miR-184. Here, the effect of CARM1 was shown on reducing cell cycle arrest at the G2/M phase and promoting migration, which is consistent with the opposing effects of miR-184 on these responses in HCECs. *LASP1* is another identified miR-184 gene target that has essential roles in controlling cell structure, physiological processes, and cell signaling. LASP1 downregulation delays HCEC migration, indicating cytoskeletal architecture involvement in miR-184 mediated biological processes. However, a more definitive accounting of the role of miR-184 in mouse corneal epithelial renewal in vivo requires additional clarification.

Current evidence has demonstrated that specific inhibition of a candidate miRNA can be achieved through antisense oligonucleotides or sponge RNA molecules [32]. This makes it possible to develop therapeutic avenues by targeting candidate miRNAs [33]. For example, using an antisense oligonucleotide blocking miR-122 showed a potent anti-HCV (hepatitis C virus) effect in clinical trials [34]. A recent study revealed that a chemically modified miR-92a inhibitor enhances angiogenesis and cutaneous wound healing [35]. Therefore, targeting miR-184 by an antisense inhibitor may be a promising avenue for promoting CEWH in the future.

## Conclusions

In conclusion, we showed that miR-184 is a critical negative regulator in CEWH, both in vivo and in vitro. We also showed there is a reciprocal relationship between increases in miR-184 expression levels and declines in migratory and proliferative activity in corneal epithelial cells. We further reveal that miR-184 target genes include *CDC25A*, *CARM1*, and *LASP1*, are essential for controlling cell proliferation and migration. Therefore, our study indicates that miR-184 can serve as a biomarker of CEWH progression and it is a potential drug target in the treatment of this process.

## Supplementary information

**Additional file 1 Supplementary Table S1.** siRNA sequences of CDC25A, LASP1, CARM1, and negative control.

**Additional file 2 Supplementary Table S2.** GO enrichment analysis of 104 differentially downregulated genes, which are also putative target genes of miR-184.

**Additional file 3 Supplementary Table S3.** A list of 93 GO terms of interest.

## Data Availability

All data generated or analyzed during this study are included in this published article and its supplementary information files.

## Abbreviations

miRNA: MicroRNA;
CEWH: Corneal epithelial wound healing
HCEC: Human corneal epithelial cell
MTS: 3-(4,5-dimethylthiazol-2-yl)-5-(3-carboxymethoxyphenyl) -2-(4-sulfophenyl)-2H-tetrazolium, inner salt
PEI: Polyethyleneimine
EGF: Epidermal growth factor
NC: Negative control
RT-qPCR: Quantitative RT-PCR
GAPDH: Glyceraldehyde-3-phosphate dehydrogenase
PI: Propidium iodide
GO: Gene Ontology
d: Day
